# Raman spectroscopy for grading of live osteosarcoma cells

**DOI:** 10.1186/s13287-015-0074-5

**Published:** 2015-04-18

**Authors:** Yi-Hung Chiang, Stewart H Wu, Yi-Chun Kuo, How-Foo Chen, Arthur Chiou, Oscar K Lee

**Affiliations:** Institute of Clinical Medicine, National Yang-Ming University, No. 155, Sec2, Linong Street, Taipei, 112 Taiwan; Department of Orthopaedics, National Yang-Ming University Hospital, No. 152, Xinmin Road, Yi-Lan, 260 Taiwan; Institute of Biophotonics, National Yang-Ming University, No. 155, Sec2, Linong Street, Taipei, 112 Taiwan; Department of Orthopaedic Surgery, Taipei City Hospital, No. 145, Zhengzhou Road, Taipei, 10341 Taiwan; Stem Cell Research Center, National Yang-Ming University, No. 155, Sec2, Linong Street, Taipei, 112 Taiwan; Department of Medical Research, Taipei Veterans General Hospital, No. 201, Sec 2, Shipai Road, Taipei, 11217 Taiwan

## Abstract

**Introduction:**

Osteosarcoma is the most common primary malignant bone tumor, and the grading of osteosarcoma cells relies on traditional histopathology and molecular biology methods, which require RNA extraction, protein isolation and immunohistological staining. All these methods require cell isolation, lysis or fixation, which is time-consuming and requires certain amount of tumor specimen. In this study, we report the use of Raman spectroscopy for grading of malignant osteosarcoma cells.

**Methods:**

We demonstrate that, based on the detection of differential production of mineral species, Raman spectroscopy can be used as a live cell analyzer to accurately assess the grades of osteosarcoma cells by evaluating their mineralization levels. Mineralization level was assessed by measuring amount of hydroxyapatite (HA), which is highly expressed in mature osteoblasts, but not in poorly differentiated osteosarcoma cell or mesenchymal stem cells, the putative cell-of-origin of osteosarcoma.

**Results:**

We found that under Raman spectroscopy, the level of HA production was high in MG-63 cells, which are low-grade. Moreover, hydroxyapatite production was low in high-grade osteosarcoma cells such as 143B and SaOS2 cells (p < 0.05). Matrix metalloproteinase MMP2, MMP9 were highly expressed in SaOS2, 143B and MSCs and decreased in human fetal osteoblast (FOB) and MG-63 cells as expected (p < 0.05). These results may highlight the inverse correlation between HA level and prognosis of osteosarcoma.

**Conclusions:**

The use of Raman spectroscopy for the measurement of HA production by the protocol reported in this study may serve as a useful tool to rapidly and accurately assess the degree of malignancy in osteosarcoma cells in a label-free manner. Such application may shorten the period of pathological diagnosis and may benefit patients who are inflicted with osteosarcoma.

## Introduction

Osteosarcoma is the most common primary malignant bone tumor and is most prevalent among children and teenagers. Osteosarcoma is defined as a malignant tumor of connective tissue origin. Malignant transformation of mesenchymal stem cells (MSCs) or osteoblastic progenitor cells during bone remodeling has been reported [1-6]. Patients with nonmetastatic osteosarcoma often have a 5-year survival rate of around 60% [7-9]. However, patients with lung metastases and poor response to chemotherapy end up with a low survival rate of 20% [2,3]. Histologic grading in osteosarcomas is therefore important in the diagnosis. For osteosarcoma, however, traditional histopathology methods are time consuming, and they can only offer semiquantitative or nonquantitative information. A sensitive and objective method for diagnosis of osteosarcoma is not readily available.

MSCs have been identified as the nonhematopoietic stem cells residing in bone marrow stoma, which have the capability of differentiation into tissues of mesodermal origin such as osteoblasts, adipocytes, chondrocytes, and tenocytes [10-13]. MSCs play an important role in normal bone formation and remodeling. Potential clinical applications of MSCs have been reported in recent years [9,14,15]. Osteoblasts, the progenies of MSCs, are bone-forming cells which are pivotal in homeostasis of the bone marrow microenvironment [16].

Raman spectroscopy has been extensively used in a wide variety of biological applications. Owing to its high sensitivity and selectivity, Raman spectroscopy has been recognized as a powerful tool and has been widely used for dynamic chemical analysis in molecular identification and drug screening [17-21]. The technique provides a detailed molecular structure, chemical composition, and molecular interaction in tissues and cells [17,18,21-23]. The molecular composition and structural characteristics in the spectra are often associated with disease severity. Hence, quantitative spectral changes specific to a particular state of disease can be sufficiently used as biomarkers [24]. Previously, we reported the differences between Raman spectra of the undifferentiated and differentiated human MSCs and demonstrated that Raman spectroscopy is an effective biosensor to monitor the production of different mineralized matrices during osteogenic differentiation of MSCs, which can be used to evaluate their maturation level of osteogenic differentiation [25]. Recently, the feasibility of using cellular Raman spectroscopic fingerprinting of cells for clinical diagnosis has been demonstrated successfully [26-28]. Importantly, MSCs have been reported as the putative cell of origin for osteosarcoma [29].

Hydroxyapatite (HA) is a natural mineral form of calcium apatite with chemical formula Ca_10_(PO_4_)_6_(OH)_2_. The mineral distribution increases with maturation upon osteoblast differentiation of MSCs [25]. We reason that it may be possible to use production of the HA molecule to detect the degree of malignancy of osteosarcoma cells, because it is known that the more malignant the cancer cells, the more immature they will be and the less HA these cells will produce [30].

The purpose of this study is to investigate the possibility of using Raman spectroscopy in the measurement of HA production to identify the degree of malignancy of osteosarcoma cells. In this study, we seek to compare the level of HA production of osteosarcoma cells [28,31] including SaOS2 and143B cells, which are high-grade osteosarcoma cells, and MG63 cells [32-34], which are low grade. Human MSCs and human fetal osteoblast (hFOB) cells serve as a reference for bone formation and are used as controls in this study. Our hypothesis is that osteosarcoma cells with different degrees of malignancy can be distinguished by the amount of HA production under Raman spectroscopy.

## Methods

### Maintenance and expansion of mesenchymal stem cells

Commercially available human MSCs were purchased from Lonza (Walkersville, MD, USA). Their ability to differentiate into osteoblasts, chondrocytes, and adipocytes was confirmed. hFOB cells, the differentiated osteoblasts, were used as controls. MG63, SaOS2 and 143B cells were obtained from Sigma-Aldrich (St. Louis, MO, USA). As adherent cells reached approximately 50 to 70% confluence, they were detached with 0.25% trypsin–ethylenediamine tetraacetic acid (Gibco, Grand Island, NY, USA), washed twice with phosphate-buffered saline (PBS; Sigma-Aldrich, St. Louis, MO, USA ), centrifuged under 1,000 rpm (200 × *g*) for 5 minutes, and reseeded at 1:3 under the same culture conditions.

### Culture medium

Expansion medium for MSCs consists of MSC growth medium (MesenPRO; Gibco), 100 U penicillin, 1,000 U streptomycin, and 2 mM l-glutamine (Gibco). Expansion medium for MG63 (osteoblast-like osteosarcoma cell line), SaOS2 and 143B (high-grade osteosarcoma cell line), and hFOB cells consists of Iscove modified Dulbecco medium (Gibco) and 10% fetal bovine serum (Hyclone, Logan, UT, USA) supplemented with 100 U penicillin, 1,000 U streptomycin, and 2 mM l-glutamine (Gibco).

### Raman spectroscopy

Raman spectra were equipped with a liquid-nitrogen-cooled detector. An 80 cm focal length spectrometer system (HR800; Jobin Yvon, Longjumeau Cedex, France) was used in this study with a 1,800 g/mm holographic grating to provide a spectral resolution of 1 cm^−1^. Spectra were recorded from 800 to 3,500 cm^−1^ with a resolution of approximately 5 cm^−1^.

MSCs were seeded on a 2 mm × 2 mm quartz coverslip and treated with osteogenic induction medium consisting of 10 mM β-glycerophosphate, 0.2 mM ascorbic acid, and 0.1 μm dexamethasone. Before measurement, cells were twice washed with PBS , and the coverslip was placed on a quartz slide. An O-ring was placed between the coverslip and the slide. The minimized chamber was filled with PBS to prevent cell death during the measurement.

Each Raman spectrum represented the average of five different replicates on one single cell surface. Labspec 5.0 software (HORIBA Scientific, Ediso, NJ, USA) was used for signal processing. All spectra in this study were normalized to CH_2_ wag at 1,449 cm^−1^.

The signal from extracellular matrix protein was measured from each spectrum, which facilitates calculation of the mineral-to-matrix ratio to indicate the relative level of mineralization in the extracellular matrix [35]. Phenylalanine, which is ubiquitously present within all extracellular matrices and with a background spectrum at 1,004 cm^−1^, was used for calculation of the mineral-to-matrix ratio of the substrates [36,37]. The band area intensity for HA in the cells was normalized against phenylalanine before averaging [23].

### Histochemical staining of hydroxyapatite

To evaluate the production of HA, cells were rinsed twice with PBS, fixed with 3.7% formaldehyde for 20 minutes, and washed with distilled water. The mineralization matrix was analyzed with Von kossa staining using 1% silver nitrate (Sigma-Aldrich) under UV light for 45 minutes, followed by 3% sodium thiosulfate (Sigma-Aldrich) for 5 minutes, and then counterstained with van Gieson (Sigma-Aldrich) for 5 minutes. The cultures were then stained for 45 minutes with 2% Alizarin red S at room temperature with shaking. Alizarin red S, a dye that stains calcium salts selectively and is widely used for mineral histochemistry of calcium, served to analyze the mineralization level of cells. The cultures were washed three times with water after staining. Stained cultures were photographed using an inverted microscope.

### Quantitative real-time PCR analysis

RNA was prepared from 3 × 10^5^*in vitro* culture cells, including human MSCs, hFOBs, and MG63, and total RNA was isolated using Trizol (Invitrogen, Grand Island, NY, USA) and cleaned using an RNA easy minikit (Quiagen, Courtaboeuf, France). We reverse transcribed the messenger RNA to complementary DNA using reagents (Genemark Technology, Taipei, Taiwan) according to the manufacturer’s instructions. Quantitative real-time PCR analysis of total RNA from cultured cells was performed using the ABI Step One Plus Real Time PCR System (Applied Biosystems, Foster City, California, USA). cDNA was amplified using an ABI Step One Plus Real Time PCR System at 95°C for 60 seconds, 56°C for 45 seconds, and 72°C for 60 seconds for 40 cycles, after initial denaturation at 95°C for 5 minutes. The primers used for amplification were: matrix metalloproteinase (MMP)2, forward 5′-TGAAGCACAGCAGGTCTCAG-3′ and reverse 5′-GTGTTCAAACCAGGCACCTC-3′; MMP9, forward 5′-GAACCAATCTCACCGACAGG-3′ and reverse 5′-GCCACCCGAGTGTAACCATA-3′; extracellular matrix metalloproteinase inducer (EMMPRIN), forward 5′-GAATGACAGCGCCACAGAG-3′ and reverse 5′-TACTCTCCCCACTGGTCGTC-3′; osteocalcin, forward 5′-TGAGAGCCCTCACACTCCTC-3′ and reverse 5′-ACCTTTGCTGGACTCTGCAC-3′; and glyceraldehyde 3-phosphate dehydrogenase, forward 5′-AGCCACATCGCTCAGACAC-3′ and reverse 5′-GCCCAATACGACCAAATCC-3′.

### Data analysis

Statistical analyses were performed by Student’s *t* test for two groups of data and by one-way analysis of variance with *post hoc* tests for multiple comparisons. Data were expressed as mean ± standard error of the mean from three independent experiments and *P* <0.05 was considered statistically significant.

## Results

### Setup of Raman spectroscopy

**Figure 1:**
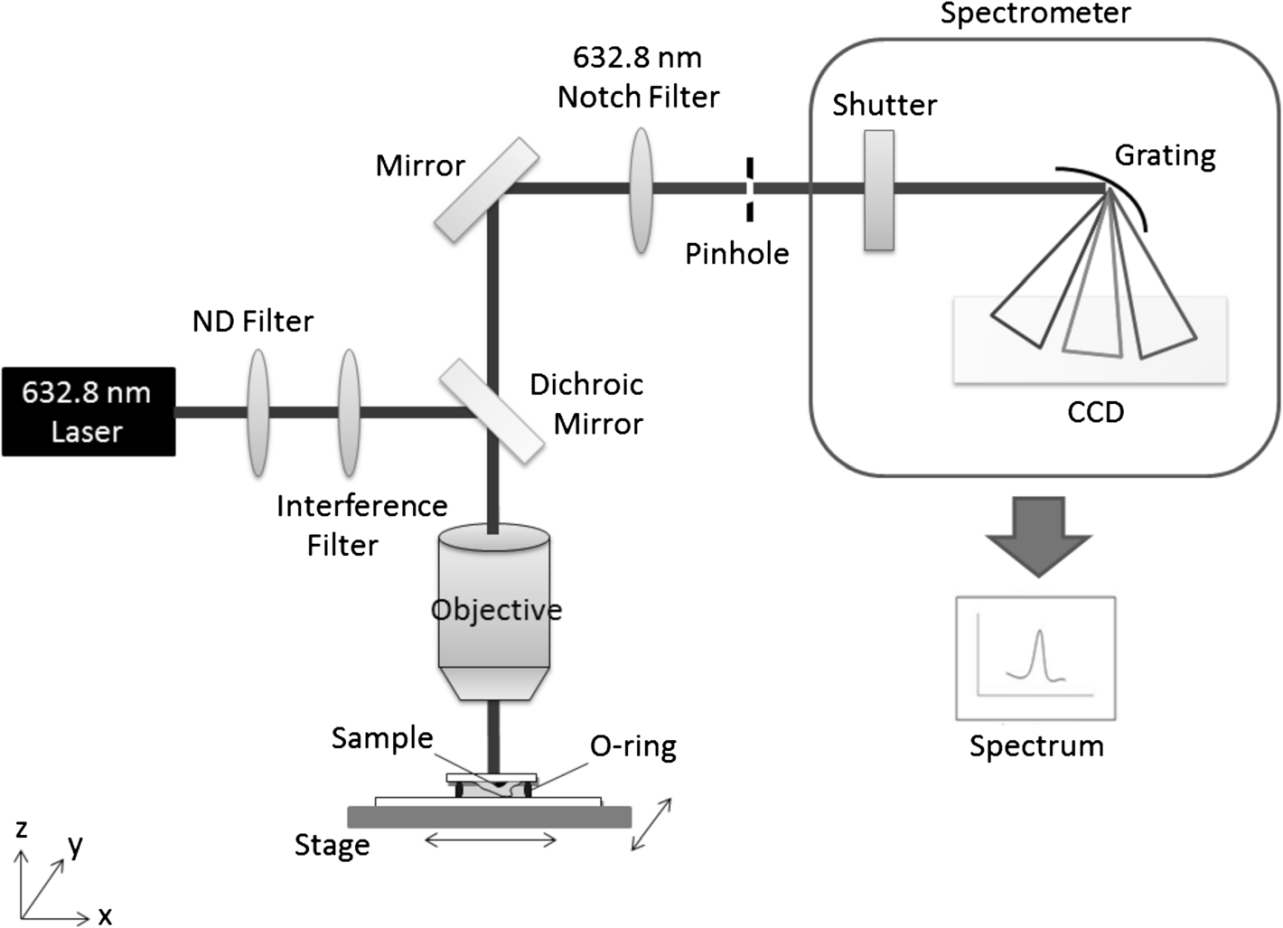
Schematic diagram of the Raman platform setup. CCD, charge-coupled device; ND, neutral density filter.

Cells were seeded on a 2 mm × 2 mm quartz coverslip and then were placed on a quartz slide. An O-ring was placed between the coverslip and the slide. In this study, an 18 mW He–Ne laser operating at 632.8 nm was used to provide the Raman excitation and the excitation beam traveled through the Neutral density filter and interference filter in order to block unwanted light and to transmit the desired wavelength. The laser power (18 mW full-beam power at the sample) was adjusted to attain a sufficient signal-to-noise ratio and to avoid sample damage. The selected laser beam then stimulated Raman effects in the sample on the quartz. For Raman spectroscopic imaging, a confocal Raman microscope (BX-41; Olympus, Shinjuku, Tokyo, Japan) was used with a 60× water immersion M-Plan objective (NA = 0.9). The objective had a 2 mm working distance to ensure minimum invasion of the cells. The spatial resolution was about 1 to 2 μm for a 1 to 2 μm laser spot size. A liquid-nitrogen-cooled, charge-coupled, two-dimensional array detector was used to measure the Raman signal by integrating for 180 seconds. Spectra were recorded from 800 to 3,500 cm^−1^ with a resolution of approximately 5 cm^−1^. A schematic diagram of the Raman spectroscopy setup is shown in Figure 1.

### Maintenance of cells

**Figure 2:**
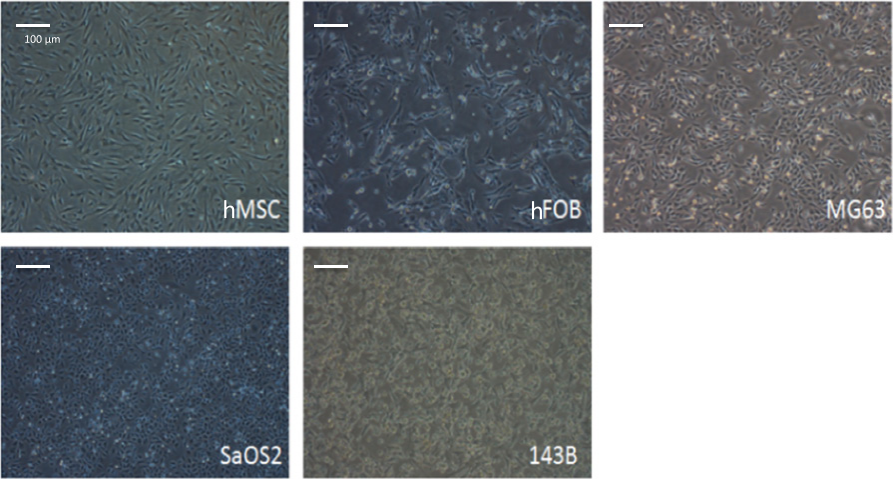
Morphology of different osteosarcoma cells. Morphology of human bone marrow-derived mesenchymal stem cells (hMSC), human fetal osteoblast (hFOB) cells, and osteosarcoma (MG63, SaOS2, and 143B) cells, at 80% confluence, under a light microscope. Magnification: 10 × .

The five different cell lines (MSC, hFOB, MG63, SaOS2, and 143B) were well maintained in culture with proper medium. The morphology of cells was observed under an optical microscope with a 10× objective 72 hours after seeding. These cells were indistinguishable in their morphology, including shape, density, and cell–cell contact. Representative photomicrographs showing the morphology of these cells are shown in Figure 2.

### Raman spectrum of different osteosarcoma cells

**Figure 3:**
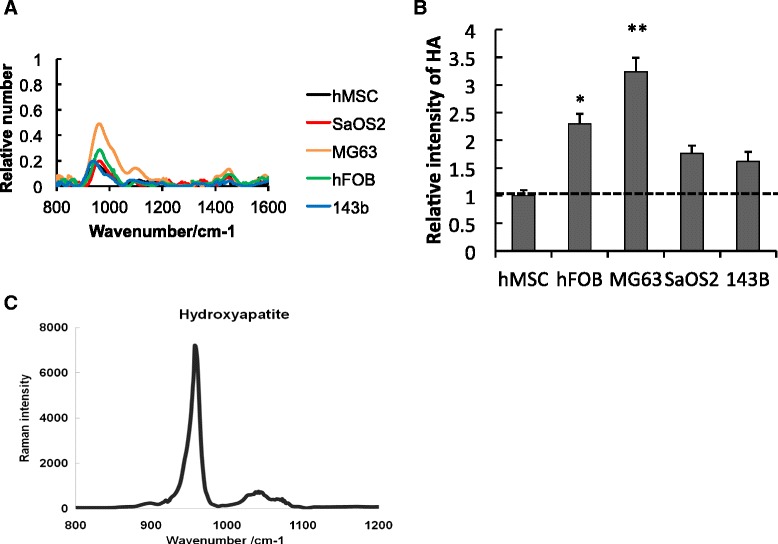
Raman spectrum of different osteosarcoma cells. **(A).** Raman spectra (averaged over different parts of the cell samples) showing the expression level of hydroxyapatite (HA) in different cells. **(B)** Raman spectra revealing significant differences in the peak intensity at the symmetric P–O stretch band (around 960 cm^−1^) of HA in different grades of osteosarcoma cells (SaOS2, 143B, and MG63). HA was highly expressed in MG63 cells, but not in human mesenchymal stem cells (hMSC) and SaOS2 and 143B cells. *P <0.05, **P <0.01. **(C)** Raman spectrum showing the symmetric P–O stretch band (around 960 cm^−1^) of HA. hFOB, human fetal osteoblast.

Raman spectra of the cells were measured and the results are shown in Figure 3A. The spectra were obtained by averaging over different locations on the cell surface with a 180-second integration time and each band envelope baseline was subtracted. Among the five cell lines, a dramatic difference was observed in the intensity of their peaks around 960 cm^−1^, which is dominated by the symmetric stretching of phosphate groups (Figure 3A,B). The band assignments for HA were confirmed by measuring the chemical powder of HA, as shown in Figure 3C. HA was highly expressed in mature osteoblasts and MG63 cells, which are low-grade osteoblast-like osteosarcoma. On the contrary, HA production was low in MSCs and highly malignant osteosarcoma cells (SaOS2 and 143B). Raman peak intensity around 960 cm^−1^ is a direct indication of the level of HA in these cells.

### Conventional biology assays for mineralization level of different cell lines

**Figure 4:**
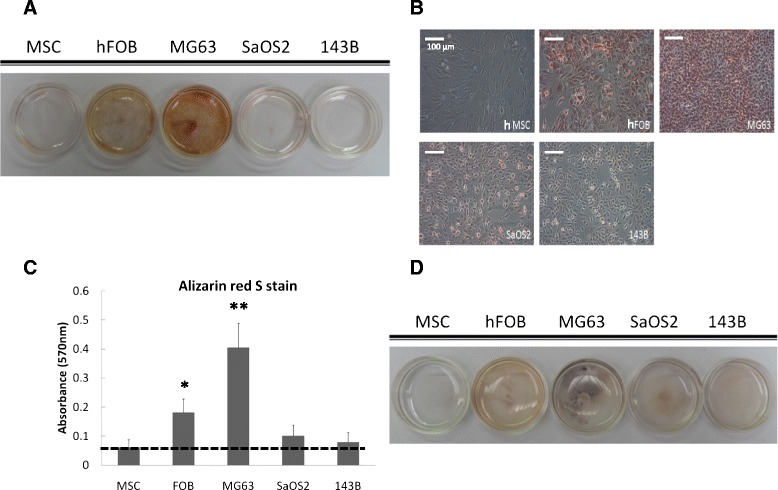
Alizarin red S staining and von Kossa staining for mineralization of different osteosarcoma cells. **(A)** Alizarin red S staining represents the mineral distribution of human bone marrow-derived mesenchymal stem cells (MSCs), human fetal osteoblast (hFOB) cells, and osteosarcoma (MG63, SaOS2, and 143B) cells. Red spot, mineralization of five cells at 80% confluence under light microscope. Magnification: 20×. **(B)** Alizarin red S staining shows mineralization spots in the MG63 and hFOB group, but not in MSCs and SaOS2 and 143B cells. **(C)** Quantification of Alizarin red S stain by the absorbance (~570 nm) of the red extracts, normalized by the number of cells in the sample. *P <0.05, **P <0.01. **(D)** Von kossa staining shows phosphate mineralization spots in the MG63 and hFOB group but not in hMSCs and SaOS2 and 143B cells.

To validate the results from the Raman spectroscopy method, Alizarin red S staining and von Kossa staining were used to evaluate the extent of mineralization. Alizarin is an organic compound that could react with calcium ions. The Alizarin red staining results (Figure 4A,B) clearly revealed that more calcium mineralization spots appeared in MG63 and hFOB cells than in hMSCs and SaOS2 and 143B cells. To quantify the calcium secretion level, the mineralization spots were extracted by 10% cetylpyridinium chloride, and the absorbance (at 570 nm) of the clear red extracts was measured; relative intensity of HA was high in hFOB and MG63 cells with statistical significance. The result is shown in Figure 4C.

The von Kossa stain (Figure 4D) showed similar features, and the results were consistent with those of the Alizarin red staining. A careful comparison of the results obtained by Raman spectroscopy with those obtained by conventional biochemical methods showed that the mineralization level obtained by the Alizarin red stain and Von Kossa stain in the culture dish did match with those obtained by Raman spectroscopy, especially the quantification of Alizarin red S stain.

### Correlation between malignancy grading and osteogenic maturation

**Figure 5:**
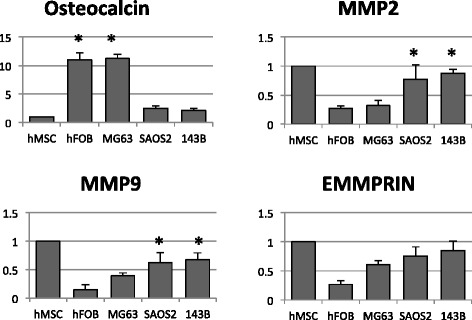
Osteocalcin gene expression level in different osteosarcoma cells. Gene expression profiles of osteocalcin, MMP2, MMP9, and EMMPRIN measured by real-time PCR and normalized by glyceraldehyde 3-phosphate dehydrogenase. *P <0.05. EMMPRIN, extracellular matrix metalloproteinase inducer; hFOB, human fetal osteoblast; MMP, matrix metalloproteinase; MSC, mesenchymal stem cell.

Real-time PCR analysis was performed to measure the osteocalcin gene expression level in these cells and the results are shown in Figure 5. Expression of osteocalcin was high in osteoblasts (hFOB cells) and low-grade osteosarcoma cells (MG63 cells). However, in undifferentiated MSCs and more malignant osteosarcoma cells such as SaOS2 and 143B, expression of osteocalcin was significantly lower.

MMP2, MMP9, and EMMPRIN are members of the MMP protein family and are involved in breakdown of the extracellular matrix in normal physiological processes, such as reproduction, embryonic development, and tissue remodeling, as well as in disease processes, such as arthritis and metastasis [38]. MMP2, MMP9, and EMMPRIN were highly expressed in SaOS2 and 143B cells and MSCs, and were decreased in hFOB and MG63 cells as expected; the levels of MMP2 and MMP9 showed statistical significance (Figure 5).

## Discussion

In this study, we demonstrate that Raman spectroscopy unequivocally distinguishes the grade of malignancy of osteosarcoma cells. Traditionally, the grading usually depends on histopathology and sometimes can be difficult. Since Raman spectroscopy provides detailed information on molecular structures, the observed spectrum at a 960 cm^−1^ peak reveals difference among different osteosarcoma cell lines, which has not been reported elsewhere.

The 960 cm^−1^ peak indicates that HA and the levels of HA are believed to be related to maturation of osteogenic differentiation. To evaluate the maturation of osteogenic differentiation, histochemical and molecular biological methods such as Alizarin red staining, von kossa staining, western blot, and RT-PCR are commonly used [39-41]. The drawback is that these assays are time consuming and detection on live cells is not possible. Previously, we have successfully developed a Raman spectroscopy-based measurement to evaluate the maturation of cells during osteogenic differentiation of MSCs and osteoblasts [25]. In this study, we further demonstrate that a similar approach can be adopted in the grading of osteosarcoma cells.

In this study, Raman spectroscopy successfully indicates the level of HA in each cell, and the RT-PCR data indicate that MMP2, MMP9, and EMMPRIN are expressed at a high level in cells with a high level of HA production. In the literature, it is reported that upregulated expression of P53, MMP, and EMMPRIN is correlated with poor prognosis in patients with osteosarcoma [38,42]. These results may highlight the inverse correlation between the HA level and prognosis of osteosarcoma.

It is apparent that conventional biochemical methods including staining and RT-PCR can distinguish different grades of osteosarcoma cells by measuring the mineralization level. However, all of these biochemical methods need cell fixation and/or cell lysis is necessary. Also, the significance of histological grading is limited by interobserver variability, in addition to the fact that the majority of tumors fall into the intermediate range, which is not easy to assess quantitatively. Raman spectroscopy has been used for clinical diagnosis of many malignant diseases including breast cancer [43], lung cancer [44], and skin cancer [45]. In this study, we also unequivocally demonstrate that Raman spectroscopy can serve as a viable real-time, quantitative, *in situ* biodetector for osteosarcoma. Our results indicated that the Raman spectroscopy technique has a high sensitivity, is easier to use to quantitatively measure the mineralization level of cells, and is more efficient than the conventional methods. Most importantly, detection via Raman spectroscopy can potentially be achieved even with a small amount of cells. From this study we advance the possibility of using Raman spectroscopy to distinguish between high-grade and low-grade osteosarcoma. The technique may theoretically therefore be further utilized in the diagnosis of other sarcomas. Taken together, the use of Raman spectroscopy may effectively facilitate the pathological diagnosis and malignancy grading of osteosarcoma.

We launched this study on the basis of cells but not tissues because Raman spectroscopy has the advantage of subcellular observation. The excited shorter wavelengths can enable subcellular resolution and extracellular water content can strongly absorb the emitted infrared light. Consequently, Raman spectroscopy can provide useful information on the molecular composition of cells. However, there are some limitations to our current Raman technique including the scan area, signal of Raman scattering, measurement conditions, and penetration depth. The scattering efficiency is quite low. The acquisition time from the cell takes several seconds, but it may take several hours to check live tissue. We are now trying to incorporate more powerful optical techniques such as surface-enhanced Raman scattering and coherent anti-Stokes Raman scattering to complement the deficits. Both techniques can amplify weak Raman signals and may complete the information for live human tissues in a much shorter time frame with greater sensitivity and accuracy. Our findings in this study can be further applied to research under surface-enhanced Raman scattering or coherent anti-Stokes Raman scattering. The level of HA can be interpreted as the level of mineralization and can be used as a label to grade the malignancy of osteosarcoma tissues. It is also possible that other biomarkers will be identified by surface-enhanced Raman scattering or coherent anti-Stokes Raman scattering. Further validation in clinical samples is warranted.

## Conclusion

Raman spectroscopy provides a precise, sensitive, and quantitative way to distinguish different grades of osteosarcoma. By targeting HA, Raman spectroscopy provides real-time and quantitative information for clinical diagnosis. This approach for the characterization of osteosarcoma cells may substantially shorten the time required for pathological diagnosis, and patients with osteosarcoma may benefit from such rapid diagnosis.

## Abbreviations

EMMPRIN: extracellular matrix metalloproteinase inducer
HA: hydroxyapatite
hFOB: human fetal osteoblast
MMP: matrix metalloproteinase
MSC: mesenchymal stem cell
PBS: phosphate-buffered saline
